# The consequence of modulating background on the luminance-response function of the human photopic electroretinogram

**DOI:** 10.1007/s10633-025-10029-y

**Published:** 2025-05-31

**Authors:** Jan Kremers, Cord Huchzermeyer, Avinash J. Aher

**Affiliations:** Department of Ophthalmology, Experimental Ophthalmology, Section for Retinal Physiology, University Hospital Erlangen, Schwabachanlage 6, 91054 Erlangen, Germany

**Keywords:** Photopic flash electroretinogram, Modulating background, Photopic hill, ERG

## Abstract

**Purpose:**

To study the consequences of a modulating background on the luminance-response function of the human photopic flash electroretinogram for different relative timings of the flash relative to the background luminance.

**Methods:**

Seven healthy subjects (age: 29–63 years; four females) participated in the study. We measured the response to flashes (9 flash strengths in total between 0.12 and 29.76 cd.s/m^2^ while doubling the strength at each step; 5 ms flash duration) on a steady background (24 cd/m^2^) and on a 1 Hz modulating background (24 cd/m^2^ mean luminance; 100% contrast). The flashes were presented at 6 different phases during the sine wave (0°, 90°, 180°, 225°, 270°, and 315°). Responses to a 1 Hz sinusoidally modulating stimulus were subtracted from the responses to the combined flash plus sine-wave stimuli to obtain the flash ERGs at different phases.

**Results:**

The a-wave and PhNR amplitudes monotonically increased with increasing flash strength. The b- and i-wave amplitudes displayed a maximum at 1.9 cd.s/m^2^, above which they decreased again (the so-called “Photopic hill effect”). The responses could be described by an addition of a logistic growth and a Gaussian. The parameters of these functions depended on the time of flash presentation relative to the background. The dependency of the a-, b- and i-wave on flash presentation time with constant flash strength could be described by a model that assumes that the amplitude depends on the Weber fraction of the flash including a delay and a saturation.

**Conclusions:**

The use of modulating backgrounds may increase the signal-to-noise ratio of flash ERGs and thus its diagnostic value. The dynamics of the response as a function of flash presentation time gives additional information about the retinal processing of flash and background. The photopic hill model allows the separation of processing in retinal On- and Off-pathways.

**Supplementary Information:**

The online version contains supplementary material available at 10.1007/s10633-025-10029-y.

## Introduction

The amplitude and implicit time of the full field electroretinographic (ERG) response to a flash depends on the flash strength. Generally, the amplitude increases with increasing flash strength. However, when presented upon a steady photopic background, the amplitude of the b-wave decreases again with increasing flash strength when the flash strength exceeds 5 to 10 cd.s/m^2^ [1]. This so-called photopic hill effect is thought to originate in the interaction between On- and Off-responses [2, 3]: whereas the On-responses depend monotonically on flash strength (the relationship of which can be described by a sigmoidal curve when plotted as a function of the logarithm of the flash strength), the Off-responses versus flash strength curve shows a maximum above which it decreases again. The amplitude of the Off-response versus the logarithm of the flash strength could be described by a Gaussian function [4] (see [5] for an alternative model). The total response is considered to be the addition of the On- and Off-responses.

In previous experiments using sinusoidally modulating instead of steady backgrounds, we found that the response amplitude of the flash ERG strongly depends on the instantaneous background luminance when the flash is presented [6]. When the flash was presented at the minimal background luminance, the responses were substantially larger compared to the responses elicited by the same flash at the maximal background luminance. Particularly the a- and b-wave amplitudes strongly depended on the instantaneous luminance. The response amplitude could be increased by a factor 3.5 for the a-wave, three for the b-wave and two for the i-wave when compared to the response amplitudes when the flash was presented upon a steady background of the same mean luminance. The increase was maximal when the luminance modulation of the background had a 1 Hz temporal frequency. The effects were smaller when 5 and 10 Hz background modulations were used. A mathematical model based on the assumption that amplitudes are proportional to the instantaneous Weber fraction of the flash including a response saturation and a delay difference between the responses to the flash and the background luminance described the relationship between stimulus strength and amplitude well. The effect was not only found in normal subjects but also in patients with retinitis pigmentosa (RP). Thus, using flashes upon a modulating background can have a substantial advantage in clinical practice because the increased responses lead to a substantially increased signal-to-noise ratio (SNR) thereby increasing the diagnostic sensitivity of the flash ERG. A further advantage of using a modulating background was that the flashes were more convenient for the subjects in comparison to flashes upon a steady background.

In the previous experiments, we employed only flashes with fixed strength. It is therefore unclear if the effects can be observed for all flash strengths. It is also unclear how the response amplitudes versus flash strength curves are altered by using a modulating background. Flashes on modulating backgrounds could be interesting for clinical applications when the response increase could be obtained for all flash strengths. Particularly, it is not clear how the photopic hill effect is influenced by the background modulation. The goal of the present study therefore was to investigate how the response amplitude versus flash strength curves were influenced when the flashes were presented upon a 1 Hz modulating background.

## Methods

### Subjects

Seven normal subjects (age: 29–63 years; four females) participated in the study. All participants were informed about the protocol and the purpose of the experiment. Informed written consent was obtained from all subjects. The experiments were conducted in accordance with the tenets of the Declaration of Helsinki and the protocol was approved by the local institutional ethics committee (medical faculty of the University Erlangen-Nürnberg). All participants underwent a comprehensive ophthalmological investigation including best-corrected visual acuity, slit-lamp examination, fundoscopy, standard automated perimetry, and pupillometry before the ERG recordings. All participants also had a normal colour vision as established with anomaloscope. The ERG recordings were performed on the same day as the ophthalmological investigation.

### ERG recordings

Monocular ERGs were recorded from the right eyes using the RETIport system (Roland Consult, Germany). The pupils were dilated with a drop of 0.5% tropicamide (Pharma Stulln GmbH, Stulln, Germany). The left eyes were occluded using an eyepatch during the recordings. A fibre electrode [7] placed over the lower conjunctiva and attached close to the inner and outer cantus, served as the active electrode. Two gold cup electrodes filled with an electrode paste (Ten20 conductive, D. O. Weaver & Co., Aurora, Colorado, USA) placed at the ipsilateral temple and on the forehead, after the skin was cleaned with Nuprep abrasive skin preparing gel (D. O. Weaver & Co.), served as reference and ground electrodes, respectively. The impedance of the electrodes was maintained below 5 KΩ. Subjects were asked to fixate at the central red LED in the Ganzfeld stimulator (Q450SC, Roland Consult). Signals were amplified (10^6^ times), bandpass filtered between 1 and 300 Hz, and sampled at 2048 Hz. For each stimulus condition, ERGs were averaged over 80 epochs, each lasting one second. The responses to the initial two seconds of each trial were discarded to avoid onset artifacts.

### Visual stimulation

The visual stimuli were created using a Ganzfeld stimulator (Q450SC; Roland Consult GmbH, Brandenburg, Germany). The Ganzfeld bowl was equipped with six different LED arrays (primaries), each with a different wavelength. In the current study, we used a combination of white, green, orange, blue and red LEDs to achieve the desired flash strengths. The visual stimuli were created by importing CSV files, that described the output (in cd/m^2^) of each LED array in one millisecond steps, into the RETIport system (Roland Consult). A Minolta LS-110 photometer was used to check the LED outputs.

Three types of visual stimuli were used in the present study. We measured the responses to:

(A) 9 different flashes with 5 ms duration and luminances between 23.25 and 5952 cd/m^2^ (strengths between 0.12 and 29.76 cd.s/m^2^) differing by a factor of two on a steady background (24 cd/m^2^). The different flash strengths were presented in a randomized sequence.
(B) a 1 Hz sinusoidally modulating stimulus (24 cd/m^2^ mean luminance; 100% contrast).
(C) the same flashes as in (A) with the same modulating background as in (B). The technique of presenting a flash stimulus on a sinusoidally modulating background is explained in details elsewhere [6]. The flashes were presented at 6 different phases during the sine wave (0°, 90°, 180°, 225°, 270°, and 315°) background, where the luminance at 0° and 180° equalled the mean luminance; the luminance was maximal (and twice the mean luminance) at 90° and zero at 270°. We included the stimuli with the flashes at 225° and 315° because we found previously that the ERGs strongly depended on stimulus phases when they were presented in the range between 180° and 360° [6]. Measurements at a certain phase were completed before another phase was presented. The sequence of flash strengths within a phase was randomized. The sequence of flash phases was also randomized.

The mean chromaticity was white with CIE1931 coordinates x = 0.3223 and y = 0.3199.

### Data analysis

The responses to the 1 Hz sine wave were subtracted from the responses to the combined flash plus sine-wave stimuli to obtain the flash ERGs at the different phases. The amplitudes and implicit times for the a-wave, b-wave, i-wave, and photopic negative response (PhNR) components were extracted from these responses and from the responses to the flashes upon the steady background.

**Fig. 1:**
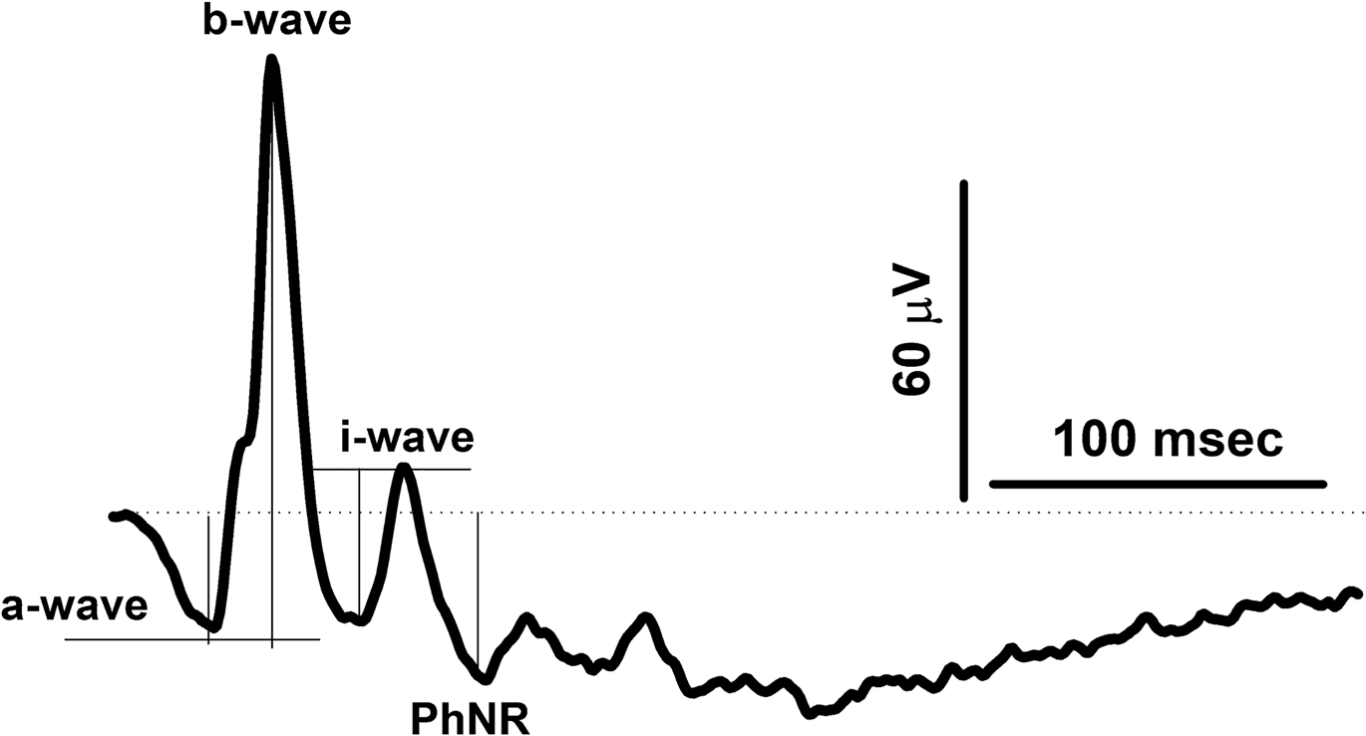
A representative flash ERG waveform on a steady background with defined ERG components

The baseline was defined as an average of potentials for the first 5 ms before the flash. Figure 1 shows a representative flash ERG elicited by 186 cd/m^2^ flash on a steady background and includes the definition of the ERG components. The leftmost point in the trace coincides with the onset of the flash. The a-wave was defined as the minimum in a time window between 12 and 19 ms after flash onset and its amplitude was measured relative to baseline. The b-wave was defined as the maximum between 24 and 35 ms after flash and its amplitude was measured relative to the a-wave trough. The i-wave was the maximum between 47 and 64 ms post-flash and the amplitudes was measured from the preceding minimum. The PhNR was defined as minimum following the i-wave and its amplitude was measured relative to the baseline. Furthermore, we evaluated the dependency of amplitudes and implicit times of ERG components on the flash strength and on the flash phase relative to the sinusoidally modulating background.

Component amplitudes versus flash phase relative to the background luminance were fitted with a model that was described previously [6]. Briefly, the model assumes that the amplitudes were proportional to the instantaneous Weber fraction *W(P)* (i.e. flash luminance divided by the instantaneous background luminance), where *P* is the phase of the flash relative to the modulation in the background (with *P* = 0° or 180° when the background luminance equals the mean luminance with positive and negative slope respectively; at *P* = 90°, the background luminance is maximal and twice the mean luminance; the background luminance is 0 cd/m^2^ when *P* equals 270°). The model includes a saturation that was described by a Naka-Rushton function ($\frac{{R_{max} \, \cdot \,W\left( P \right)}}{W\left( P \right) + b}$; *R*_*max*_ is the maximal response; *b* is the Weber fraction for half maximal response; we previously had a noise component,* C*, included which was not used here because inclusion did not improve the fits substantially but it could assume unrealistic values). The saturation resulted in realistic response amplitudes at very high Weber fractions (the Weber fraction at 270° is infinite). As a result, the response was always maximal when the Weber fraction was also maximal. In the model, a phase delay between maximal response to and maximal Weber fraction was included. This delay was considered to be the processing time of the background information in the response to the flash [6].

B- and i-wave amplitudes as a function of flash strength were fitted with a model previously described by Hamilton et al. [4]. This model assumes that the response amplitudes can be described by an addition of a Naka-Rushton function and of a Gaussian function. The Naka-Rushton function can be described by $\frac{{V_{max} \, \cdot \,I}}{{I + I_{0.5} }}$, where *I* is the flash strength, V_*max*_ is the maximal response and *I*_*0.5*_ is the flash strength for half maximal response; we used different designations of the these parameters to distinguish them from the Naka-Rushton used for the fits of the amplitude versus phase data). The mathematical description of the Gaussian function is: $G_{max} \, \cdot \,e^{{ - \left( {\frac{{\left( {logI - \mu } \right)}}{\sigma }} \right)^{2} }}$ where *G*_*max*_, *μ* and *σ* are the maximal value, mean and standard deviation of the Gaussian respectively. Whereas the Naka-Rushton function uses flash intensity (*I*) as the independent variable, the Gaussian function uses its logarithm. The Naka-Rushton and Gaussian functions are assumed to describe the response amplitudes of the On- and the Off-pathways respectively [4]. In the fits, there were five free parameters (*V*_*max*_, *I*_*0.5*_, *G*_*max*_, *μ* and *σ*). The model was successfully implemented for describing intensity-response functions including the photopic hill in several studies [8, 9] and is included in an extended protocol of the International Society for Clinical Electrophysiology of Vision (ISCEV) [10].

## Results

**Fig. 2:**
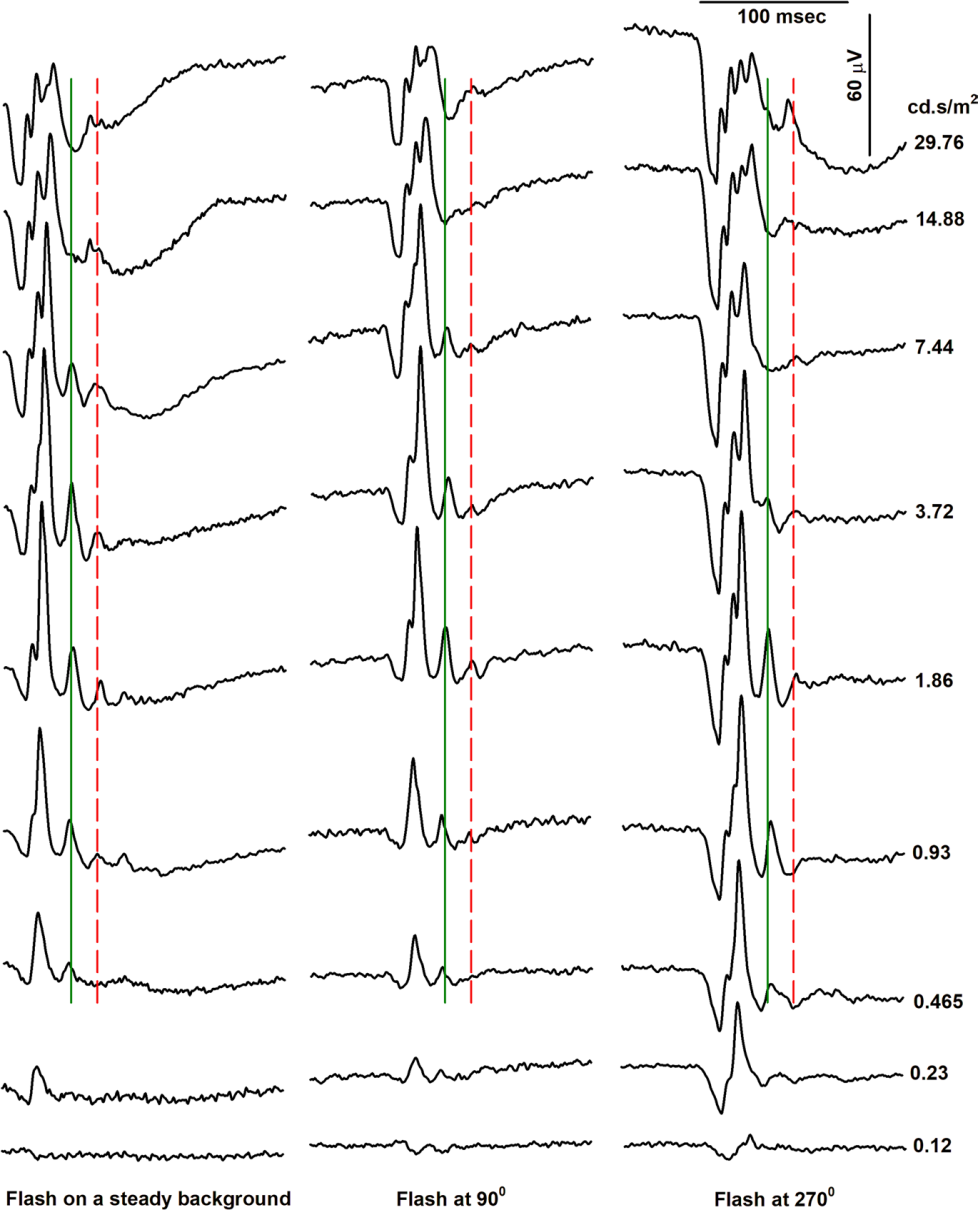
The representative original flash ERG waveforms on a steady background (left), pulse presented at 90° on sinusoidally modulating background (middle), and the pulse presented at 270° (right) respectively for increasing flash strengths from bottom to top

Figure 2 shows the original ERG waveforms recorded from a representative subject for all flash strengths. For the sake of clarity, we have presented here only the waveforms recorded on a steady background (left) and at two [90° (middle) and 270°(right)] phases during the sinusoidally modulating background. The employed flash strength that elicited the responses doubled from the bottom to top. For all three conditions, the ERG waveform changed with increasing flash strength. In addition, the ERG responses to flashes upon a modulating background strongly depended on the phase of flash presentation. The responses to flashes presented at 270° were substantially larger than those to flashes at 90° and upon a steady background, particularly with weaker flashes. The differences were smaller with strong flashes. In all three conditions, the largest response was obtained with 1.86 or 3.72 cd.s/m^2^ flashes.

### a-wave

**Fig. 3:**
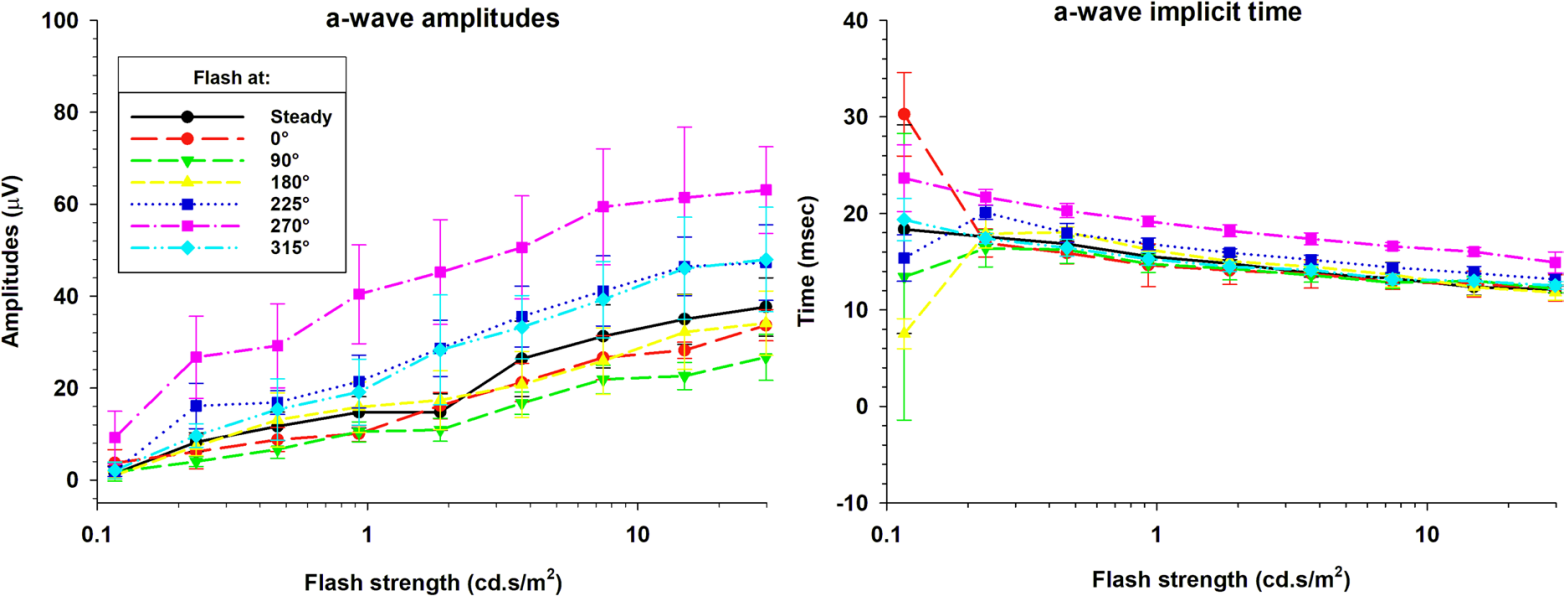
The group averaged a-wave amplitudes (left panel) and implicit times (right panel) as a function of flash strength in logarithmic scale. The graph shows data recorded with a steady background (black trace) and six different phases on the sine wave background (incrementing colours)

Figure 3 presents the a-wave (mean ± 1 s.d.) amplitudes (left panel) and implicit times (right panel) as a function of flash strength and plotted separately for the different phases of the flashes relative to the background and when a steady background was used. With increasing flash strength, the a-wave amplitudes increased monotonically. The a-wave amplitudes were larger than those obtained with a steady background for the flashes presented at 270° and smaller for the flashes at 90°. The maximal responses were three to four times larger than the minimal responses. The a-wave amplitudes for the flashes presented upon a steady background were similar to those that were elicited by flashes presented at 0° and 180° relative to the sine wave background, i.e. when the sine wave and the steady backgrounds had identical instantaneous luminances, confirming our previous results [6]. The a-wave amplitudes with flashes at 225° and 315° were also larger than those obtained with a steady background.

The a-wave implicit times were variable with the weakest flashes that hardly elicited a response for certain conditions (see also Fig. 2). For flashes at 270°, the responses were measurable (see also Fig. 2) and meaningful implicit times were obtained. In general, the implicit times decreased with increasing flash strengths. This can be observed for all conditions and confirms previous findings [3, 11]. The a-wave implicit times were slightly higher for the flashes presented at 270° again confirming previous results [6].

**Fig. 4:**
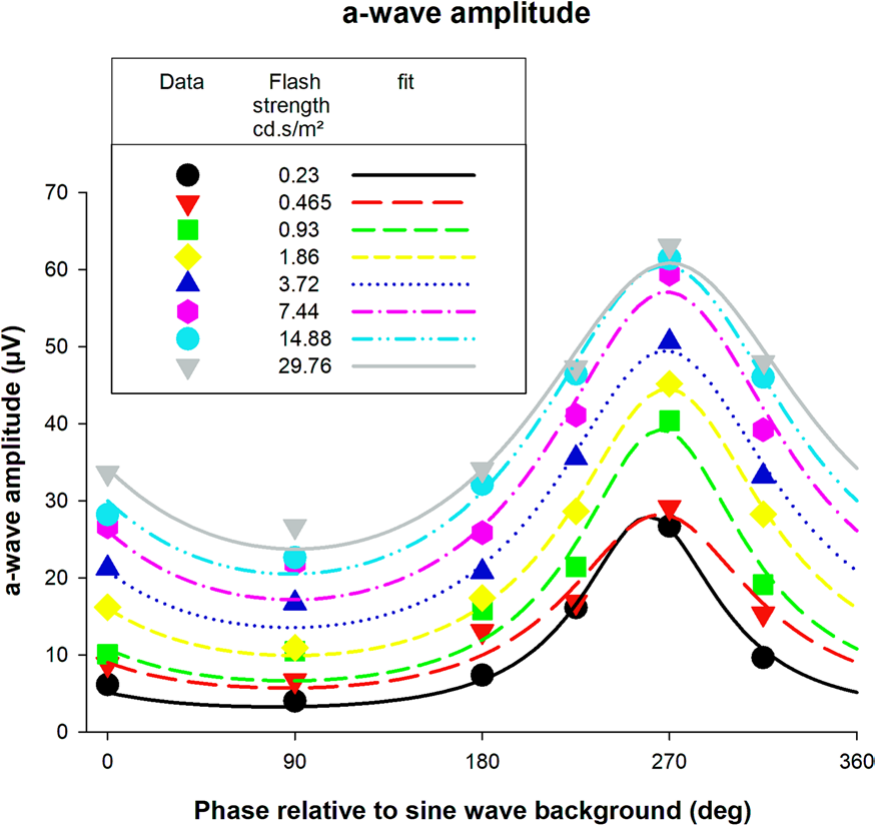
A-wave amplitudes of ERG responses elicited by flashes upon a sine wave modulating background. The amplitudes are plotted as a function of the phase during the sine wave at which the flash was presentation. The different symbols represent data for the different flash strengths. The curves are fits of a previously described model [6] to the data

Figure 4 displays the a-wave amplitudes elicited by the flashes upon the sine wave background as a function of the phase at which the flash was presented, plotted separately for the different flash strengths (for clarity we did not show error bars; these can be obtained from the left plot in Fig. 3; this is also the case for similar plots for the other response components). As the weakest flashes did not elicit a measurable response in most conditions, the data obtained with these stimuli are not shown. For all flashes strengths, the a-waves were largest when the flashes were presented at about 270° where the luminance of the background was 0 cd/m^2^. The in Fig. 4 curves are fits of the model that we introduced previously [6] and that is described briefly in the Methods section to the data. Clearly the model can describe the data satisfactorily.

**Fig. 5:**
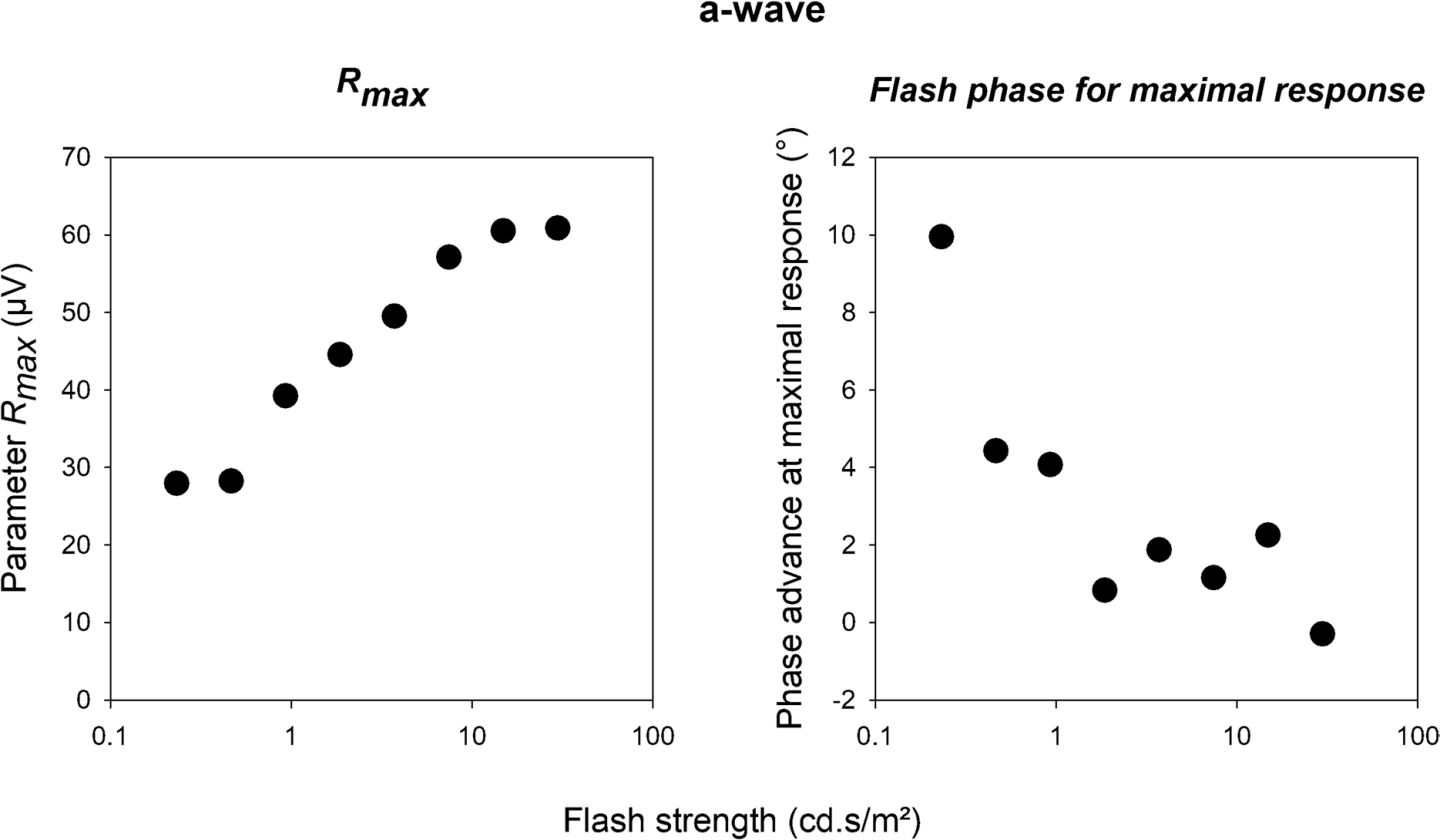
*R*_*max*_ and phase advance of the flashes for a maximal response derived from the fits through a-wave amplitude versus flash phase data displayed in Fig. 4. By definition, positive phase values indicate a phase advancement, where the flashes had to be delivered before the background luminance was minimal (and the Weber fraction was maximal) for a maximal response

From the model fits we obtained the *R*_*max*_ and flash phase advances for a maximal response. These are shown in Fig. 5 as a function of flash strength. The *R*_*max*_ values increased with increasing flash intensity which is not surprising as the overall response increased. The phases were generally small but the data were nevertheless consistent. Two aspects are of interest. First, the phases were positive, indicating that the flash had to be presented before the background reached the minimum of zero cd/m^2^ (and Weber fraction was maximal), indicating a phase advance. This indicates that not only the instantaneous background luminance is of importance. An additional differentiator that results in a phase advancement should be considered. Second, the phase advancement decreased with increasing flash strengths suggesting that the above-mentioned differentiator is mainly of importance with weak flashes. This result is seemingly in contrast to the finding that the a-wave implicit time decreases with increasing flash strength (Fig. 3 right plot). However, the phases were based on amplitude data and are thought to reflect the time needed to process the background luminance rather than flash intensity.

### b-wave

**Fig. 6:**
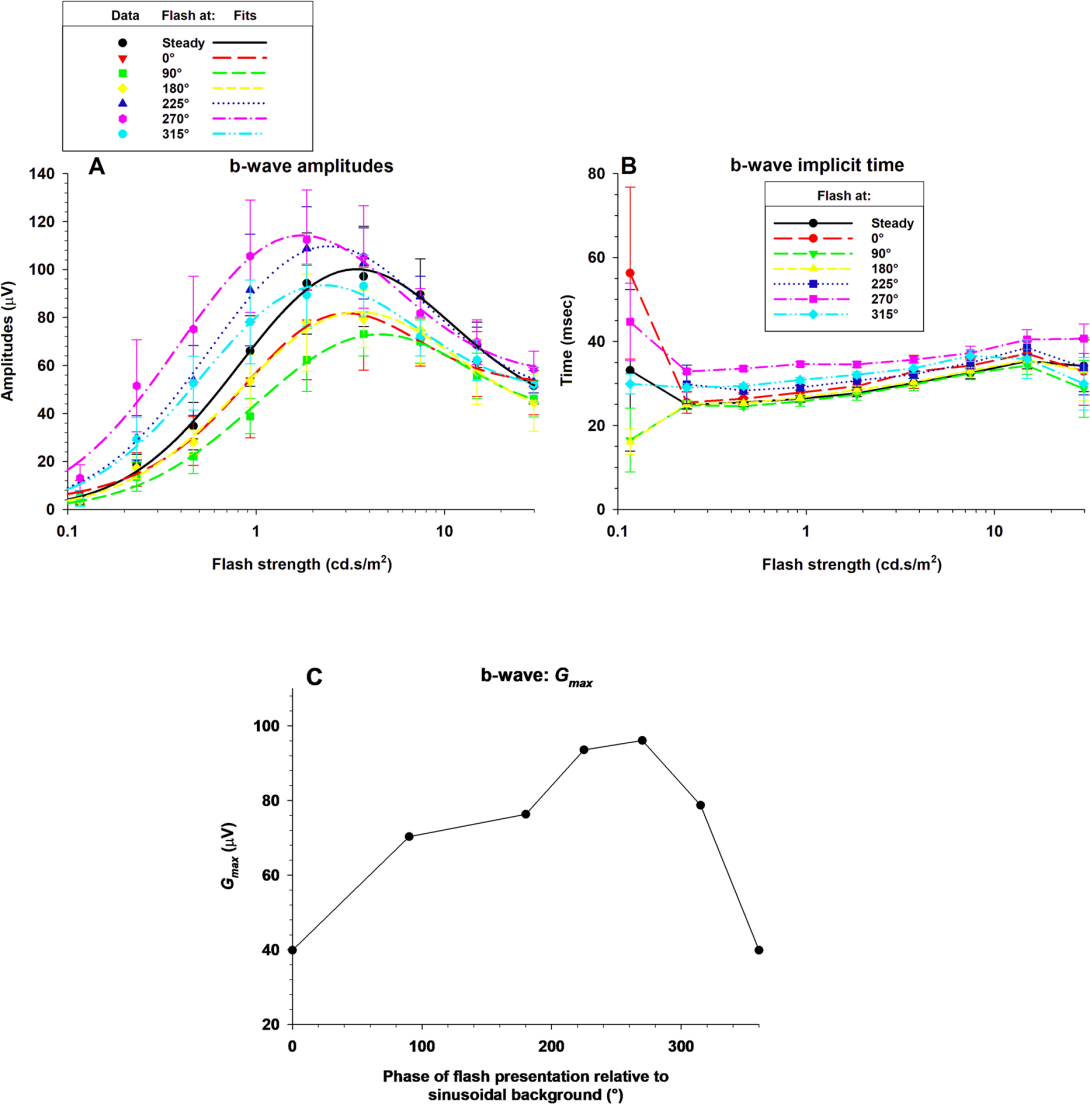
The group averaged b-wave amplitudes (**A**) and implicit times (**B**) as a function of flash strength on a logarithmic scale. The data are displayed separately for the responses on a steady background and for the different phases relative to the sinusoidally modulated background. The curves show the fits of the model of Hamilton et al. [4] to the data. **C**: The values of *G*_*max*_*,*, obtained from the model fits, given versus phase of flash presentation relative to the 1 Hz sine wave background

Figure 6 shows the group averaged (± 1 s.d.) b-wave amplitudes (A) and implicit times (B) as a function of flash luminance. The b-wave amplitudes for both steady and sine wave background increased with increasing flash strength until it reached a maximum with flashes between 1.5 and 5 cd.s/m^2^ (i.e. between 300 and 1000 cd/m^2^) above which the amplitudes decreased again, indicative for the photopic hill. The maximal b-wave amplitude varied between about 75 µV and 110 µV for flashes presented at 90° and 270°, respectively. For weaker flashes, the responses with flashes at 270° were up to four times larger than those presented at 90°. Beyond the photopic hill, the response decrease depended on the time of flash presentation and was largest for flashes at 270°. As a result, the b-wave amplitudes were similar at the strongest flashes.

The model of Hamilton et al. [4] (see also the description in the Methods section) were fitted to the data. The model could describe the data well. In the fits, the data did constrain the parameters of the Gaussian function in the model well. The *G*_*max*_ values are of particular interest because they quantify the magnitude of photopic hill effect. They are given as a function of phase of flash presentation in Fig. 6C. The photopic hill effect was largest with flash presentations close to the minimum of the background sine wave. The Naka-Rushton functions were less well constrained by the data.

The b-wave implicit times increased with increasing flash strength by about 5 ms, but they did not further increase (or even decreased slightly) with the strongest flashes. This result is in agreement with previous results [3, 11]. The implicit times were a few milliseconds longer when the flash was presented at 270° of the sine-wave background. In all other conditions they were similar.

**Fig. 7:**
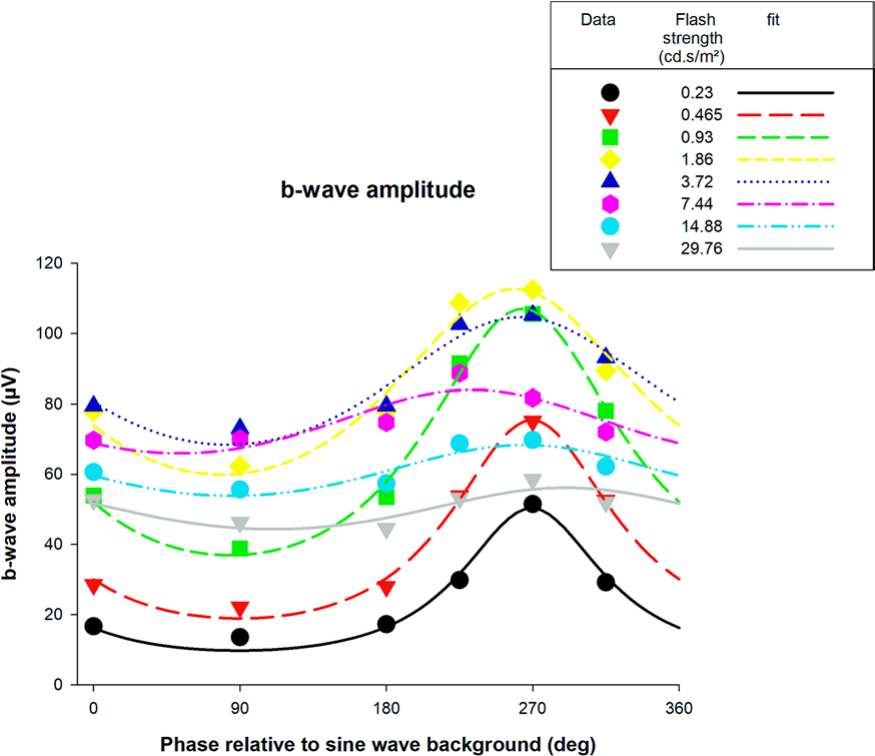
B-wave amplitudes of flash ERGs with sine wave background plotted against phase relative to the background and fitted with the function described in the text. Separate plots and fits are shown for the different flash strengths

The b-wave amplitudes of the responses obtained with sine wave backgrounds were given as a function of the phase and fitted with the same function as used for the a-waves. The results are shown in Fig. 7. The dependency of the b-wave amplitudes on the phase was large with weak flashes, and clear maxima could be observed. Beyond to the photopic hill (flashes stronger than 3.72 cd.s/m^2^), the response did not depend as strongly on phase. Overall, the model for the a-wave could also describe the b-wave amplitudes satisfactorily.

**Fig. 8:**
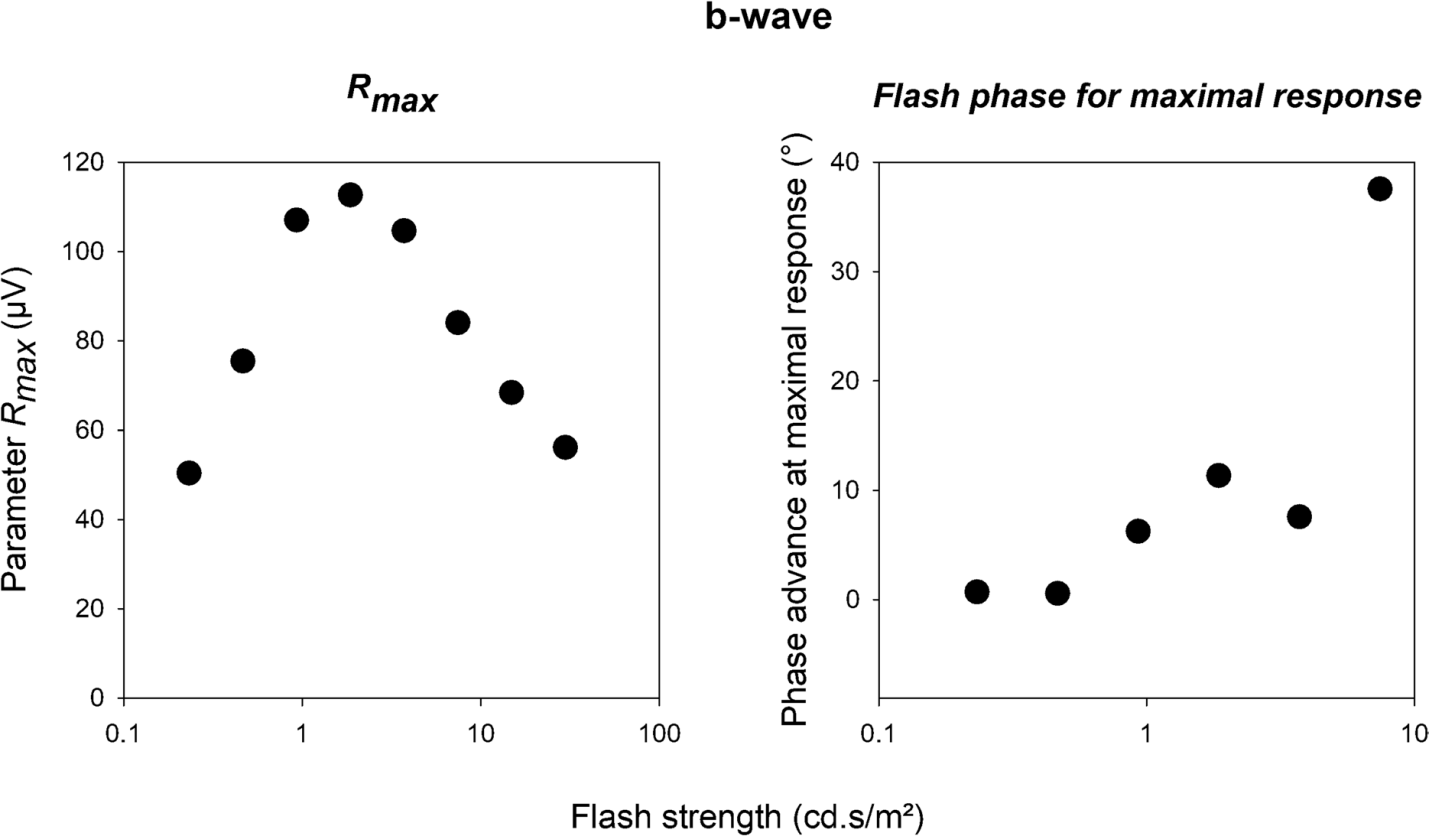
R_max_ and phase advance of the flashes for a maximal response derived from the fits through b-wave amplitude data displayed in Fig. 7

Similar as for the a-wave, we plotted *R*_*max*_ and flash phase for maximal response against flash strength (see Fig. 8). Since the modulation was very weak at the two highest flash strengths, the phases were not well constrained by the data. Therefore, they were discarded. The *R*_*max*_ values clearly show a photopic hill effect. In contrast to the a-wave, the phase at maximal response increased with increasing flash strength, indicating that the phase advance of the maximal response increases with increasing flash strength.

### i-wave

**Fig. 9:**
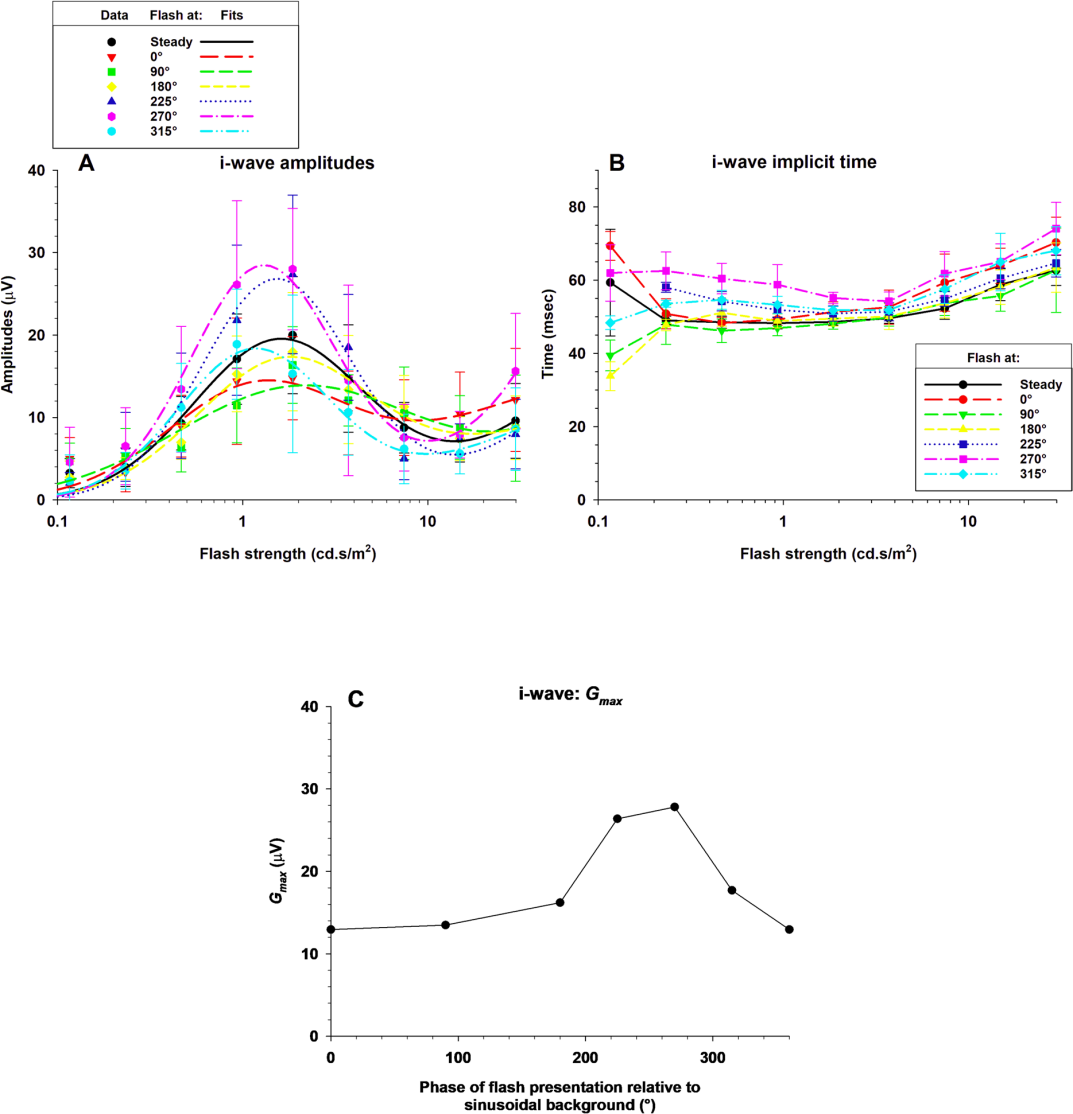
The group averaged i-wave amplitudes (**A**) and implicit times (**B**) as a function of flash strength. The graph shows data recorded with a steady background (black trace) and six different phases on the sine wave background (incrementing colours). **C**: The values of *G*_*max*_, obtained from the model fits, given versus phase of flash presentation relative to the 1 Hz sine wave background

Figure 9 depicts the group averaged i-wave amplitudes (A) and implicit times (B). The i-wave amplitude and time data were more variable than those of the a- and b-waves. However, clearly the i-wave amplitudes also showed a strong photopic hill effect. This is in agreement with data obtained by Rufiange et al. [3]. They were maximal at flash intensities of about 300 cd/m^2^ above which they decreased. Then they displayed a minimum at about 15 cd.s/m^2^ above which they increased again. We fitted the amplitude data with the same model that was used for describing the b-wave amplitude. The model could describe the i-wave data well. We found that the Gaussian (thought to originate in the Off-pathway) described the amplitudes up to the minimum at about 15 cd.s/m^2^. The Naka-Rushton function (supposed to reflect activity of the On-pathway) mainly described the data at the two highest flash intensities. Figure 9C depicts the values of *G*_*max*_, obtained from the model fits, to the data with modulating backgrounds, plotted as a function of the flash phase. Again, the photopic hill effect was largest when the flashes were presented close to the luminance minimum of the sine wave background.

The implicit time initially did not change strongly or decreased slightly. At higher intensities (i.e. where the amplitudes increase again) the implicit times increased by up to 20 ms. As can be seen in the original responses shown in Fig. 2, there are two separate peaks that can be assigned as i-wave (indicated by the drawn black and dashed red vertical lines). Two i-waves were also identified by Rufiange et al. [3]. At flash strengths up to the amplitude minimum the analysis captured the first peak (indicated by the drawn black lines in Fig. 2). At higher flash strengths the analysis probably mainly captured the second peak with larger implicit times (indicated by the dashed red lines in Fig. 2).

**Fig. 10:**
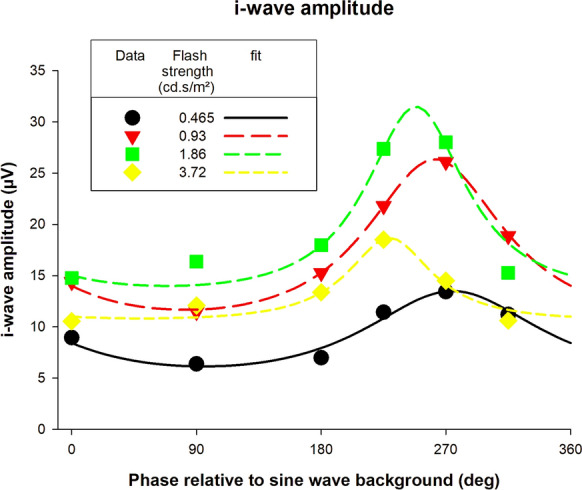
I-wave amplitudes of flash ERGs with sine wave background plotted against phase relative to the background and fitted with the function described in the text. Separate plots and fits are shown for the different flash strengths at 0.465, 0.93, 1.86 and 3.72 cd.s/m^2^ where substantial i-waves could be measured for all phases

**Fig. 11:**
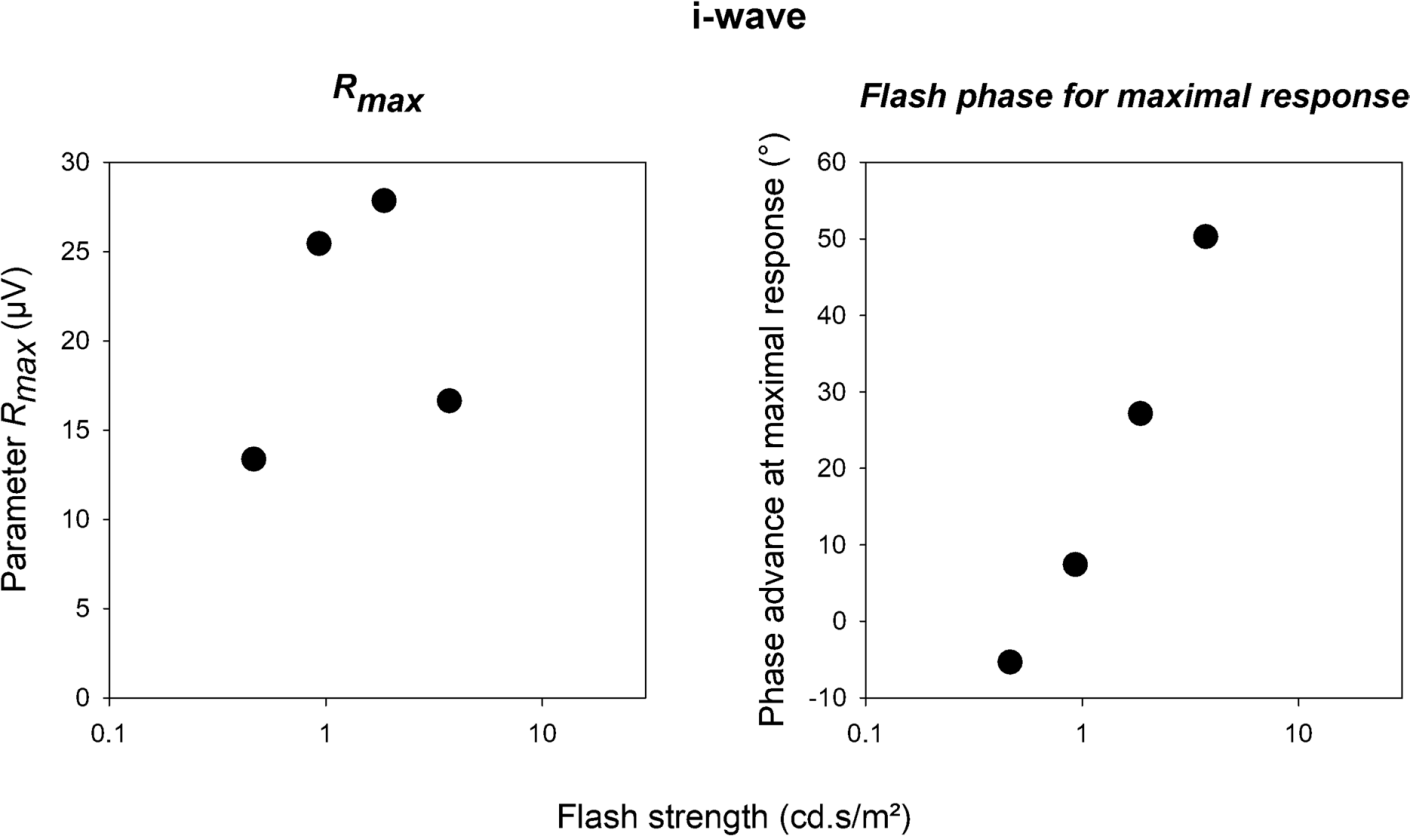
R_max_ and phases of the flashes for a maximal response derived from the fits through i-wave amplitude data displayed in Fig. 10

The data in Fig. 9 also show that the response amplitude was influenced by the phase relative to the background at flash strengths between about 0.465 and 3.72 cd.s/m^2^. The largest i-wave amplitudes were obtained with flashes presented at 270° or 225°. The i-wave responses to flashes on a steady background were larger than during the bright phase of the sine wave background. For the mean luminances between 0.465 and 3.72 cd.s/m^2^, we plotted the i-wave amplitudes as a function of the flash phase. The results are shown in Fig. 10. Again, the model could describe the phase dependency of the i-wave amplitudes (curves in Fig. 10) satisfactorily. The influence of the phase of flash presentation was, however, smaller as for the a- and b-waves. The *R*_*max*_ and phase parameters obtained from these fits are plotted in Fig. 11. Since the model was only applied at four mean luminances, there was only a limited number of data. However, the *R*_*max*_ again showed a photopic hill and the phase advance strongly increased with increasing mean luminance.

### PhNR

Group averaged PhNR amplitudes (left panel) and implicit times (right panel) are shown in supplementary material Fig. 1. The PhNR data were quite variable. However, the PhNR amplitudes did not show a photopic hill. The PhNR implicit times did not exhibit a strong dependency on flash luminance but tended to increase with increasing flash strength for flashes above about 5 cd.s/m^2^.

In Supplementary material Fig. 2, the PhNR amplitudes are plotted as a function of phase of the flash relative to the sine wave background for the different mean luminances. The amplitudes were larger when the flashes were delivered at the background luminance troughs (i.e. at phases around 270°), but the effects were less clear than with the other components. In addition, as mentioned above, the PhNR data were much more variable. We therefore did not fit these data.

## Discussion

The purpose of the current study was to provide a description of the ERG responses to flashes with different intensities when presented upon a sinusoidally modulating background. We previously found that a modulating background could have a strong influence on ERGs elicited by flashes of 0.5 cd.s/m^2^ (500 cd/m^2^; 1 ms) strength [6]. We found that the influence was particularly large when the background modulated at a 1 Hz temporal frequency with 100% Michelson contrast. We therefore used this background modulation for the present study.

### Photopic hill

We found that the a-wave and the PhNR increased monotonically with flash strength whereas the amplitudes of the b- and i-waves decreased when elicited by very strong flashes, known as the photopic hill effect. The photopic hill was described and modelled before [1–4, 9, 11, 12]. Wali and Leguire [1] found that the effect is not affected if the flashes are presented in an order of increasing or decreasing strength. We presented the flashes in a randomized order so that the influence of the presentation sequence was minimized. In addition, we did not notice any effect if the flashes were preceded by weaker or stronger flashes. We are therefore confident that we obtained photopic hill effects similar to those presented previously.

In the present study, maximal b-wave amplitudes were obtained with flashes between 1 and 5 cd.s/m^2^. This agrees well with the results reported by Rufiange et al. [3] and Hamilton et al. [4]. Hamilton et al. also found that the b-wave implicit time decreased beyond the photopic hill. We, however, found that the implicit times increased further. A comparison of the original responses of the present (Fig. 2) and their (their Fig. 2) studies shows that the responses in our study displayed larger oscillatory potentials (OPs) than theirs, which possibly influenced the implicit times. The cause of this difference is unclear. The OP amplitudes strongly depended on flash strength (see Fig. 2). In our previous study [6] we used relatively weak flash (0.5 cd.s/m^2^) explaining why the OPs were relatively small in that study.

All b-wave amplitude versus flash intensity curves could be described by the model introduced by Hamilton et al. [4], which consists of a Gaussian function and a logistic growth described by a Naka-Rushton equation. The Gaussian function is thought to describe Off-responses whereas the Naka-Rushton function is considered to describe the responses in the On-pathway [2, 3]. We found that the photopic hill effect (described by the Gaussian function) increased when flashes were presented at a phase of about 270° relative to the background (i.e. when the background was dim; Fig. 6C). An influence of the background luminance on the response to the flash, with larger responses when the background luminance was lower was described before by Rufiange et al. [3]. They also found that the background luminance can have an effect on the photopic hill. In that respect, our data are in agreement with theirs. However, it should be noted that Rufiange et al. measured with steady backgrounds after sufficient adaptation time, resulting in a steady state. We used a modulating background where the luminance was continuously changing. This dynamic aspect of the background may explain differences between theirs and ours results. For instance, Rufiange et al. found that the maximal b-wave amplitude was similar for all backgrounds whereas we found that the maximal b-wave amplitude varied depending on the instantaneous background luminance. Another aspect, where the dynamics of the background show an influence, concerns the b- and i-wave amplitudes to flashes presented at 0° and 180° phase. They were smaller than those with the steady background although the instantaneous luminances were identical in these three measurements. We propose that not only the background luminance but also the change in the background luminance (i.e., the slopes of the luminance waveforms given by the first derivative) may influence the b- and i-waves in the flash responses. This may also be the cause for the differences between the responses at 0° and 180° phases (particularly visible for the i-waves; see Fig. 9A) because the slope was positive at 0° and negative at 180°. This dynamic aspect may also be the cause for the phase advancement for a maximal response (Figs. 7, 10). The origin of this dynamic mechanism has to be determined yet. However, it seems to be unlikely that conventional adaptation mechanisms are involved, because the time constant of the dynamic aspect is in the range of msecs whereas adaptation effects have time constants in the range of secs and minutes. Particularly rod intrusion is unlikely to play a role. It was found previously that rod signals could be detected up to 400 td retinal illuminance [13]. With a dilated pupil this would be equivalent to 8 cd/m^2^ luminance. We calculated that during a period of about 267 ms the luminance of the background is below this level (observe that this is an approximation because differences in mean chromaticity have been ignored). We think that this period is too short for rod signals to recover and intrude the flash ERGs. The b-wave morphology is also characteristic for a photopic flash ERG. A modulatory effect of rod driven signals on the photopic ERG cannot be excluded though. Immediate effects, possibly with an origin in the rods, after extinction of the background are described before [14]. In agreement with this finding, rod-driven signals (isolated with the silent substitution method) in mouse ERGs were found to adapt slowly but changes in their amplitude and timing could be observed immediately after a step-wise change in background luminance [15]. Further measurements are needed to firmly establish a similar rod-driven effect on the photopic hill phenomenon in human subjects.

The b-wave amplitudes to very strong flashes did not strongly depend on the phase of the flash occurrence. This indicates that the responses of the On- and Off-pathways in the b-wave response are similarly influenced by the modulation of the background luminance so that their effects cancel each other out.

We also found a strong photopic hill effect in the i-wave amplitudes again in agreement with the data from Rufiange et al. [3]. The i-wave (with an implicit time of about 55 m) can be exclusively described by the Gaussian function. The photopic hill was again maximal for flashes that were presented when the luminance of the background was minimal (Fig. 9C). Assuming that, as for the b-wave, the photopic hill is a feature of the Off-pathway, this indicates that the i-wave is mainly an Off-response. This proposition agrees with findings that the i-wave disappears when the Off-pathways is blocked with PDA [16]. We also found a positive component with an implicit time of about 70 ms. This component is mainly discernible in the responses to the strongest flashes but partially it occurred together with the i-wave. The amplitudes of the component could be described by a Naka-Rushton function. We are reluctant, however, to contribute this to an On-pathway response because this component was quite variable. Additional studies would be necessary to establish if the two components are related.

The photopic hill effects for the b- and i-waves is possibly correlated with other described effects where On- and Off-pathways have different influence on the measured responses. This may include an negative flash ERG, a separation of responses to luminance increments and decrements (using long flashes or stimuli with sawtooth temporal profiles) [17, 18] and the responses to luminance sine-waves that show a conspicuous minimum at about 12 Hz, which is thought to originate in the cancelling interaction between On- and Off-responses [19]. It has been shown that in patients where the On- and Off-pathways are differently affected (such as CSNB, X-linked retinoschisis and Duchenne Muscular Dystrophy), the minima at 12 Hz in the responses to luminance sine waves are less obvious than in normal subjects [12, 17, 20]. As a result, responses to 12 Hz red-green heterochromatic modulation possibly reflect the luminance content of the stimulus in these patients, whereas they reflect the chromatic content in normal control subjects [18, 21]. It has been shown that CSNB patients display an increased photopic hill because of the absence of an On-pathway [4, 9, 12]. It remains to be established if DMD and X-linked retinoschisis patients show the same effect.

### Response amplitude versus time of flash presentation

In our previous study, we found that modulating backgrounds influenced the a- and b-waves more strongly than the i-wave and the PhNR. The present study confirmed this observation. The largest a-wave was between two and five times larger than the smallest (Fig. 4); the maximal b-wave amplitudes was up to a factor of about three larger than the minimal b-wave amplitude (Fig. 7), whereas i-wave and PhNR could be maximally enhanced by a factor of two (Figs. 10 and 13). The largest ratio between minimal and maximal amplitude was found for weaker flashes (in the range between 40 and 400 cd/m^2^). The origins of components with larger implicit times probably are located in more inner retinal structures [22] where more elaborate signal processing may occur. We propose that an increased temporal integration of the signals generated by the background results in a decreased influence of the instantaneous background luminance.

Another feature that we found was that the flash phase for a maximal response depended on flash intensity: while the phase decreased (i.e. the response became less advance) with increasing flash intensity for the a-wave (Fig. 5, right plot), the phases increased for the b- and i-waves (right plots of Figs. 8, 11 respectively). Furthermore, the phase change was larger in the later components. For the a-wave, the phase was maximally 10° (28 ms relative to the 1 Hz background at flash intensities between 46.5 and 5952 cd/m^2^). For the b- and i-wave, the phase was maximally 40° (about 110 ms, flash intensity range: 46.5–1488 cd/m^2^) and about 65° (about 180 ms, flash intensity range: 93–744 cd/m^2^) respectively. The slopes showed these differences more clearly: assuming a linear relationship between phase and log(I), the slopes were -3.57, 7.12 and 61.99°/log(I) for the a-, b- and i-waves respectively. Again, it seems that additional retinal processing might be responsible for this. Since the responses become more phase advanced for later components, and this indicates that increasing numbers of differentiators are involved.

In conclusion, the present paper shows that the response to a flash can be influenced by the use of a modulating background. The response amplitudes (particularly of the a- and b-waves) are substantially larger at intermediate flash strengths. This can be interesting for clinical use because the increased amplitudes lead to higher signal-to-noise-ratios and, therefore, better diagnostic power of the ERG. Alternatively, flash strengths can be decreased (making the procedure more convenient for the patients) without losing diagnostic power.

## Supplementary Information

Below is the link to the electronic supplementary material.

Supplementary file1 (DOCX 45 kb)

Supplementary file2 (DOCX 21 kb)

## References

[1] Wali N, Leguire LE (1992) The photopic hill: a new phenomenon of the light adapted electroretinogram. Doc Ophthalmol 80:335–345 1473449 10.1007/BF00154382

[2] Kondo M, Piao CH, Tanikawa A, Horiguchi M, Terasaki H, Miyake Y (2000) Amplitude decrease of photopic ERG b-wave at higher stimulus intensities in humans. Jpn J Ophthalmol 44:20–28 10698021 10.1016/s0021-5155(99)00172-0

[3] Rufiange M, Rousseau S, Dembinska O, Lachapelle P (2002) Cone-dominated ERG luminance-response function: the Photopic Hill revisited. Doc Ophthalmol 104:231–248 12076014 10.1023/a:1015265812018

[4] Hamilton R, Bees MA, Chaplin CA, McCulloch DL (2007) The luminance-response function of the human photopic electroretinogram: a mathematical model. Vis Res 47:2968–2972 17889925 10.1016/j.visres.2007.04.020

[5] Akula JD, Ambrosio L, Howard FI, Hansen RM, Fulton AB (2019) Extracting the ON and OFF contributions to the full-field photopic flash electroretinogram using summed growth curves. Exp Eye Res 189:107827 31600486 10.1016/j.exer.2019.107827 PMC6956400

[6] Kremers J, Aher AJ, Huchzermeyer C (2023) Modulation of flash ERGs by dynamic backgrounds. Doc Ophthalmol 146:33–51 36266494 10.1007/s10633-022-09902-x PMC9911495

[7] Dawson WW, Trick GL, Litzkow CA (1979) Improved electrode for electroretinography. Investig Ophthalmol Vis Sci 18:988–991 478786

[8] Garon ML, Rufiange M, Hamilton R, McCulloch DL, Lachapelle P (2010) Asymmetrical growth of the photopic hill during the light adaptation effect. Doc Ophthalmol 121:177–187 20711798 10.1007/s10633-010-9243-0

[9] Garon ML, Dorfman AL, Racine J, Koenekoop RK, Little JM, Lachapelle P (2014) Estimating ON and OFF contributions to the photopic hill: normative data and clinical applications. Doc Ophthalmol 129:9–16 24894580 10.1007/s10633-014-9446-x

[10] McCulloch DL, Kondo M, Hamilton R, Lachapelle P, Messias AMV, Robson AG, Ueno S (2019) ISCEV extended protocol for the stimulus-response series for light-adapted full-field ERG. Doc Ophthalmol 138:205–215 30929108 10.1007/s10633-019-09685-8

[11] Rufiange M, Dumont M, Lachapelle P (2005) Modulation of the human photopic ERG luminance-response function with the use of chromatic stimuli. Vis Res 45:2321–2330 15924945 10.1016/j.visres.2005.02.010

[12] Rufiange M, Dassa J, Dembinska O, Koenekoop RK, Little JM, Polomeno RC, Dumont M, Chemtob S, Lachapelle P (2003) The photopic ERG luminance-response function (photopic hill): method of analysis and clinical application. Vis Res 43:1405–1412 12742110 10.1016/s0042-6989(03)00118-4

[13] Maguire J, Parry NR, Kremers J, Kommanapalli D, Murray IJ, McKeefry DJ (2016) Rod electroretinograms elicited by silent substitution stimuli from the light-adapted human eye. Transl Vis Sci Technol 5:13 27617180 10.1167/tvst.5.4.13 PMC5015991

[14] Rousseau S, Lachapelle P (1999) The electroretinogram recorded at the onset of dark-adaptation: understanding the origin of the scotopic oscillatory potentials. Doc Ophthalmol 99:135–150 11097118 10.1023/a:1002679932462

[15] Joachimsthaler A, Kremers J (2019) Mouse cones adapt fast, rods slowly in vivo. Investig Ophthalmol Vis Sci 60:2152–2164 31100107 10.1167/iovs.18-26356

[16] Rangaswamy NV, Frishman LJ, Dorotheo EU, Schiffman JS, Bahrani HM, Tang RA (2004) Photopic ERGs in patients with optic neuropathies: comparison with primate ergs after pharmacologic blockade of inner retina. Investig Ophthalmol Vis Sci 45:3827–3837 15452095 10.1167/iovs.04-0458

[17] McAnany JJ, Alexander KR, Kumar NM, Ying H, Anastasakis A, Fishman GA (2013) Electroretinographic findings in a patient with congenital stationary night blindness due to a novel NYX mutation. Ophthalmic Genet 34:167–173 23289809 10.3109/13816810.2012.743570 PMC3842226

[18] Barboni MT, Martins CM, Nagy BV, Tsai T, Damico FM, da Costa MF, de Cassia R, Pavanello M, Lourenco NC, de Cerqueira AM, Zatz M, Kremers J, Ventura DF (2016) Dystrophin is required for proper functioning of luminance and red-green cone opponent mechanisms in the human retina. Investig Ophthalmol Vis Sci 57:3581–3587 27388051 10.1167/iovs.16-19287

[19] Kondo M, Sieving PA (2001) Primate photopic sine-wave flicker ERG: vector modeling analysis of component origins using glutamate analogs. Investig Ophthalmol Vis Sci 42:305–312 11133883

[20] Barboni MTS, Zobor D, Szabo V, Besztercei B, Lesch B, Varsanyi BL, Nagy ZZ, Huchzermeyer C, Ventura DF, Kremers J (2024) Sine-wave flicker ERG changes associated with on- and off-bipolar cell asymmetries. Investig Ophthalmol Vis Sci 65:5875

[21] Zobor D, Besztercei B, Lesch B, McAnany JJ, Park JC, Barboni MTS, Kremers J (2023) Retinoschisin-1 (RS1) is required for proper functioning of luminance and red–green cone-opponent mechanisms. Investig Ophthalmol Vis Sci 64:5417 10.1167/iovs.16-19287 27388051

[22] Frishman LJ (2006) Origins of the electroretinogram. In: Heckenlively JR, Arden GB (eds) Principles and practice of clinical electrophysiology of vision. The MIT Press, Cambridge, London, pp 139–183

