# Supplementary material for: The consequence of modulating background on the luminance-response function of the human photopic electroretinogram
Source: Doc Ophthalmol. 2025 May 31;151(2):145–60. doi: 10.1007/s10633-025-10029-y (PMC12436525; doi:10.1007/s10633-025-10029-y)
Supplement: Supplementary file 1 — Supplementary file1 (DOCX 45 kb) [file 10633_2025_10029_MOESM1_ESM.docx]

Supplementary material Figure 1: Group averaged PhNR amplitudes (left panel) and implicit times (right panel) as a function of flash strength. The graph shows data recorded with a steady background (black trace) and six different phases on the sine wave background.
