# Supplementary material for: The consequence of modulating background on the luminance-response function of the human photopic electroretinogram
Source: Doc Ophthalmol. 2025 May 31;151(2):145–60. doi: 10.1007/s10633-025-10029-y (PMC12436525; doi:10.1007/s10633-025-10029-y)
Supplement: Supplementary file 2 — Supplementary file2 (DOCX 21 kb) [file 10633_2025_10029_MOESM2_ESM.docx]

Supplementary material Figure 2: PhNR amplitudes of flash ERGs plotted against phase relative to the background for the different flash strengths.
